# Supplementary material for: Silicon-Organic Hybrid (SOH) Mach-Zehnder Modulators for 100 Gbit/s on-off Keying
Source: Sci Rep. 2018 Apr 3;8:2598. doi: 10.1038/s41598-017-19061-8 (PMC5883022; doi:10.1038/s41598-017-19061-8)
Supplement: Supplementary file 1 — Supplementary Information [file 41598_2017_19061_MOESM1_ESM.pdf]

Silicon-Organic Hybrid (SOH) Mach-Zehnder Modulators for 100 Gbit/s On-Off Keying
# - Supplementary Information -

Stefan Wolf[1†], Heiner Zwickel[1†], Wladislaw Hartmann[1,2,3†], Matthias Lauermann[1,4], Yasar Kutuvantavida[1,2], Clemens Kieninger[1,2], Lars Altenhain[5], Rolf Schmid[5], Jingdong Luo[6], Alex K.-Y. Jen[6], Sebastian Randel[1], Wolfgang Freude[1], and Christian Koos[1,2,*]

[1]Institute of Photonics and Quantum Electronics (IPQ), Karlsruhe Institute of Technology (KIT), 76131 Karlsruhe, Germany
[2]Institute of Microstructure Technology (IMT), Karlsruhe Institute of Technology (KIT), 76131 Karlsruhe, Germany
[3]Now with: Physikalisches Institut, University of Muenster, 48149 Muenster, Germany
[4]Now with: Infinera Corporation, Sunnyvale, CA, United States
[5]Micram Microelectronic GmbH, 44801 Bochum, Germany
[6]Department of Chemistry, City University of Hong Kong, Kowloon, Hong Kong SAR

†these authors contributed equally to the work
email: christian.koos@kit.edu

Chirp analysis of SOH modulators

For transmission of intensity-modulated signals, an unwanted phase modulation, also referred to as chirp, leads to increased sensitivity with respect to fiber dispersion and hence leads to a signal quality penalty[1]. Ideally, SOH MZM should not exhibit any chirp – the field-induced refractive index change of the polymer cladding according to the Pockels effect[2] allows to completely suppress chirp in an ideal, perfectly balanced push-pull Mach-Zehnder modulator (MZM)[3]. For real-world devices with finite extinction ratio (ER), however, chirp is introduced by an unbalance of the optical amplitudes in the two MZM arms. For quantifying the chirp of a data signal generated by such a device, we use the chirp parameter α that is essentially defined by the ratio of the phase modulation to the amplitude modulation[4,5], and that can be estimated from a direct measurement of the static extinction ratio $\delta^{(\mathrm{stat})}$, see Equations (7) and (8) of the main manuscript. To proof that finite static extinction ratio (ER) of the MZM is the dominant source of chirp in our devices, we compare the chirp parameter obtained from the ER to a direct measurement of α. To this end, we use another SOH modulator with a device layout similar to that of the device used for the data transmission experiments, but with better static extinction ratio of $\delta^{(\mathrm{stat})} = 31$ dB.

For a direct measurement of the chirp parameter, we rely on the fiber response peak method[6]. Using a network analyzer, we measure the frequency response of the modulator with a 75 km fiber span. Erbium-doped fiber amplifiers (EDFA) are used to compensate the insertion loss of the modulator and the fiber span. The measured frequency response normalized to the back-to-back transmission is shown in Figure 1 a). For data evaluation, the product of the square of the resonance frequency f_μ and the fiber length L is plotted in dependence of the resonance order μ, see Figure 1 b). The chirp parameter α and the fiber dispersion coefficient D can then be extracted from the slope and from the vertical offset of a straight line fitted to the data points[6]. Measurement analysis leads to a dispersion coefficient D of 16.8 ps/(nm km) for the 75 km fiber span and a chirp factor α of 0.09. The value compares well to the value $|\alpha| = 0.06$ which we estimate directly from the measured (static) extinction ratio. This confirms that the chirp of SOH MZM is dominated by the influence of a finite ER – the slight differences are attributed to the finite measurement accuracies of the fiber response peak method.

Using the equations described above, we estimate a chirp factor $|\alpha| \approx 0.42$ for a static extinction ratio of 14 dB as obtained for the MZM used for the 100 Gbit/s data experiment in the manuscript, see discussion in the main manuscript.

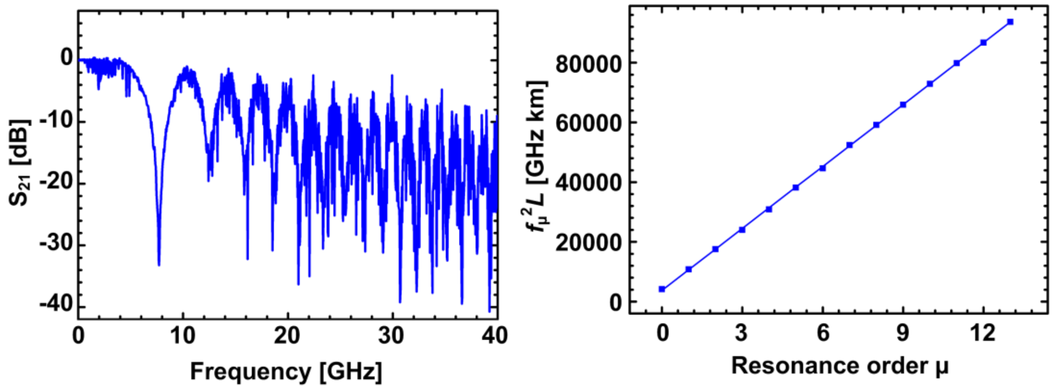

Figure 1: Chirp characterization of SOH Mach-Zehnder modulator. (a) Frequency response of SOH MZM with 75 km fiber span for modulator operated without gate field. The frequency response has been normalized to the modulator's back-to-back characterization. Resonant dips originate from fiber dispersion. (b) From the product of the squared resonance frequency and the fiber length, $f_\mu^2 L$, fiber dispersion parameter and chirp factor α can be estimated[6]. In the analysis, we find a chirp factor of $\alpha \approx 0.09$.

Wavelength operating range of SOH modulators

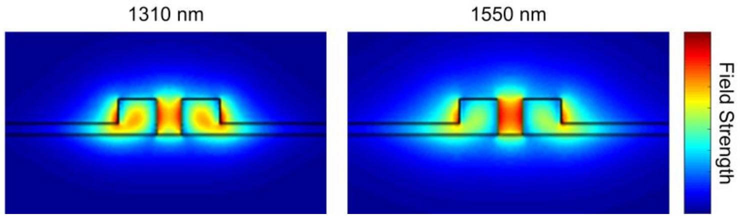

Figure 2: Simulated mode fields for a center wavelength of 1310 nm (left) and 1550 nm (right). The color coding indicates the modulus of the transverse electrical field and is identical for both plots. For the shorter wavelength, the field is stronger in the silicon rails, and hence the field interaction factor is smaller by 17 %. This is overcompensated by an increase of the material-related figure-of-merit for EO activity, $n_{EO}^3 r_{33}$.

SOH modulators can operate over a wide range of wavelengths. In essence, the operating range is only limited by the ability of the silicon photonic slot waveguide to efficiently guide the light and by the absorption of the organic EO material. The modulator structure as well as the organic material are well suited to operate over the entire range of infrared telecommunication wavelengths, comprising all relevant transmission bands between 1260 nm and 1675 nm. Absorption of the EO material is negligible above 1200 nm [7,8] and hence the material is transparent in wavelength regions beyond 1260 nm which are relevant for telecommunications. The performance of the slot waveguides can be quantified by considering the field interaction factor according to Eq. (2) of the main manuscript. For the device used in the transmission experiments of the main manuscript, the field interaction factor decreases from $\Gamma \approx 0.16$ at $\lambda = 1550$ nm to $\Gamma \approx 0.14$ at $\lambda = 1310$ nm, see Figure 2 for the associated mode field profiles. At the same time, the material-related figure-of-merit for EO activity, $n_{EO}^3 r_{33}$, increases[9] by about 40 %, which leads to an overall improvement of the modulation efficiency by about 16 %. In total, the device performance does hence not change significantly with wavelength.

Implementation of pulse shape for system simulation

The hardware used in our experiment features nonzero rise and fall times, see eye diagram in Figure 3 (a) for the measured electrical output signal. For an emulation of these signals in the simulation, we use cosine-shaped pulses in the time-domain (not to be confused with raised-cosine pulse shaping with a raised-cosine shaped spectrum). A mathematical description of the pulse shape $p(t)$ is given as

$$p(t) = \frac{1}{2}\left(1 + \cos\left(\pi \frac{t}{T}\right)\right), -T \leqslant t \leqslant T, \tag{1}$$

where T denotes the symbol duration. With the discrete symbols $a_k = \{-1;1\}$ which correspond to a logical "1" and a logical "0", respectively, with the Dirac function $\delta(t)$ representing the time-discrete nature of digital data, and with the peak-to-peak output voltage U_{pp}, the (electrical) mean-free time-domain drive signal can be written as

$$s(t) = U_{pp} \sum_k a_k \delta(t - kT) * p(t) \tag{2}$$

where $*$ denotes a convolution operation. The eye diagram of the digitally generated waveform (with additive white Gaussian noise) is depicted in Figure 3 (b).

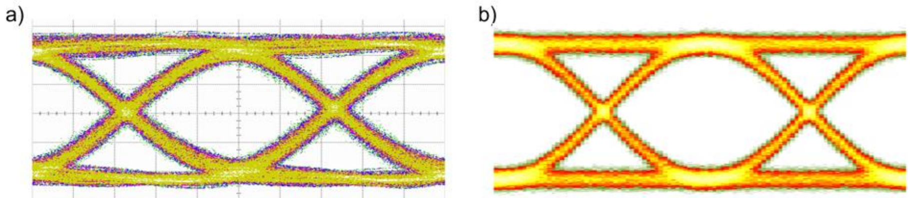

Figure 3: Waveform for bandwidth analysis. (a) Measured eye diagram from the MUX at 100 Gbit/s, having a peak-to-peak voltage swing of approximately 0.4 V_{pp}. The signal is fed to an RF amplifier and to the SOH modulator. (b) Eye diagram of digital waveform that is used to approximate the output signal of the MUX for the bandwidth simulation in the main manuscript.

References

[1] Gnauck AH, Korotky SK, Veselka JJ, Nagel J, Kemmerer CT, Minford WJ, et al. Dispersion penalty reduction using an optical modulator with adjustable chirp. *IEEE Photon Technol Lett* 1991; **3**: 916–918.

[2] Lauermann M, Wolf S, Schindler PC, Palmer R, Koeber S, Korn D, *et al.* 40 GBd 16QAM Signaling at 160 Gb/s in a Silicon-Organic Hybrid Modulator. *J Lightw Technol* 2015; **33**: 1210–1216.

[3] Walklin S, Conradi J. Effect of Mach-Zehnder modulator DC extinction ratio on residual chirp-induced dispersion in 10-Gb/s binary and AM-PSK duobinary lightwave systems. *IEEE Photon Technol Lett* 1997; **9**: 1400–1402.

[4] Kim H, Gnauck AH. Chirp characteristics of dual-drive mach-zehnder modulator with a finite DC extinction ratio. *IEEE Photon Technol Lett* 2002; **14**: 298–300.

[5] Koyama F, Oga K. Frequency chirping in external modulators. *J Lightw Technol* 1988; **6**: 87-93.

[6] Devaux F, Sorel Y, Kerdiles JF. Simple measurement of fiber dispersion and of chirp parameter of intensity modulated light emitter. *J Lightw Technol* 1993; **11**: 1937–1940.

[7] Jouane Y, Chang Y-C, Zhang D, Luo J, Jen AK-Y, Enami Y. Unprecedented highest electro-optic coefficient of 226 pm/V for electro-optic polymer/TiO2 multilayer slot waveguide modulators. *Opt Express* 2014; **22**: 27725–27732.

[8] Heni W, Haffner C, Elder DL, Tillack AF, Fedoryshyn Y, Cottier R, et al. Nonlinearities of organic electro-optic materials in nanoscale slots and implications for the optimum modulator design. *Opt Express* 2017; **25**: 2627–2653.

[9] Bosshard C, Sutter K, Schlesser R, Günter P. Electro-optic effects in molecular crystals. *J Opt Soc Am B* 1993; **10**: 867–885.
